# An evaluation of the influence of the publication of the UK National Institute for health and Care Excellence’s guidance on hypertension in pregnancy: a retrospective analysis of clinical practice

**DOI:** 10.1186/s12884-020-2780-y

**Published:** 2020-02-12

**Authors:** Diane Farrar, Derek Tuffnell, Trevor A. Sheldon

**Affiliations:** 1 Bradford Institute for Health Research, Bradford, UK; 2 Health Sciences Department, University of York, York, UK

**Keywords:** NICE hypertension in pregnancy guidance, Midwives and obstetricians perceptions, retrospective analysis, Clinician interviews

## Abstract

**Background:**

The UK National Institute for health and Care Excellence (NICE) publish guidance aimed at standardising practice. Evidence regarding how well recommendations are implemented and what clinicians think about guidance is limited. We aimed to establish the extent to which the NICE Hypertension in pregnancy (HIP) guidance has influenced care and assess clinician’s attitudes to this guidance.

**Methods:**

Five maternity units in the Midlands and North of England took part in the retrospective evaluation of 2490 birth records from randomly selected dates in 2008–2010 and 2013–2015. The proportion of women where care was adhered to before (2008–2010) and after (2013–2015) guidance publication was examined and differences estimated. Eleven midwives and obstetricians employed by Bradford Hospitals were interviewed.

**Results:**

The proportion of high risk women prescribed Aspirin rose (before 14%, after 54%, p = < 0.01 (confidence interval of change (CI) 37, 43%) as well as for moderate risk women (before 3%, after 54%, p = < 0.01, CI 48, 54%) following guidance publication. Three quarters had blood pressure and a third proteinuria measured at every antenatal visit before and after guidance. Early birth < 37 weeks and ≥ 37 weeks gestation was more frequently offered after guidance publication than before (< 37 weeks: before 9%, after 18%, *p* = 0.01, CI 2, 16% and ≥ 37 weeks before 30%, after 52%, p = < 0.01, CI 9, 35%). Few were informed of future risk of developing a hypertensive disorder or had a documented postnatal review; however there was an increase in women advised to see their GP for this review (58% before and 90% after guidance p = < 0.01, CI 24, 39%).

All clinicians said the NICE HIP guidance was informative and provided robust evidence, however they said length of the document made use in practice challenging. They did not always access NICE guidance, preferring to use local guidance at least initially; both obstetricians and midwives said they accessed NICE guidance for in-depth information.

**Conclusions:**

NICE HIP guidance is valued, used by clinicians and has influenced important aspects of care that may help improve outcomes for women who develop hypertension or pre-eclampsia, however some recommendations have had limited impact and therefore interventions are required to improve adherence.

## Background

Up to 10% of pregnancies are affected by hypertensive disorders (pre-existing hypertension, gestational hypertension and pre-eclampsia). Hypertensive disorders of pregnancy (HDP) are leading causes of perinatal morbidity and mortality, including preterm birth, small for gestational age and caesarean birth [1, 2]. Evidence suggests women exposed to gestational hypertension or pre-eclampsia are at increased risk of longer-term cardiovascular disease, Alzheimer’s disease and diabetes and all-cause mortality [3–8]. Their offspring are at increased risk of raised blood pressure, cardiovascular disease and stroke [9–11]. It is important, therefore, to try and prevent or identify hypertensive disorders early.

If implemented, evidence-based guidance can increase the quality of care, effectiveness of treatment and reduce unwarranted clinical variation [12], it is therefore important to understand the extent guidance is adhered to and what may increase or decrease use. Clinical variation occurs when differences in care cannot be explained by variation in the condition or patient preferences and can only be explained by clinical management decisions [13, 14]. The National Institute for health and Care Excellence (NICE) [15] and other similar institutions in other countries [16] have produced guidance that aims to standardise care and reduce unwarranted variation. NICE guidance recommendations are produced following the assessment of available evidence by a multidisciplinary group of individuals including clinicians and lay representatives. Clinicians are encouraged to fully consider NICE’s recommendations, alongside the woman’s needs, preferences and values when planning care.

NICE first published their guidance on the management of hypertension in pregnancy (HIP) in August 2010 [15] with an update in 2019 [17]. Recommendations include the care of women with: pre-existing or chronic hypertension (hypertension identified before the 20th week of pregnancy), gestational hypertension (hypertension identified at or after the 20th week of pregnancy) and pre-eclampsia (hypertension diagnosed at or after the 20th week of pregnancy with concurrent significant proteinuria) [15]. Guidance recommendations aim to ensure the swift identification of deviations from normal (for example blood pressure level), allowing enhanced surveillance and timely intervention [18].

NICE has been hailed as an exemplary model for health technology assessment both nationally and internationally [19, 20], however evidence regarding how well NICE guidance recommendations are implemented and clinician’s attitudes to guidance is limited. Our aims were to establish the extent to which the NICE’s HIP guidance has influenced clinical care and to understand how midwives and obstetricians view the NICE HIP guidance.

## Methods

We collected quantitative data from maternal records to examine clinical practice adherence to NICE guidance, we also collected qualitative data on clinician’s views of NICE guidance and how they used guidance in day to day practice.

### Data on clinical practice

We conducted a retrospective case note review of clinical care practice related to the identification and treatment of hypertensive disorders before (2008–2010) and after (2013–2015) the publication of the NICE HIP 2010 guidance. Routine data were unavailable for many of the aspects of care we examined. An interim period between 2011 and 2012 was excluded to allow time for dissemination and implementation of guidance recommendations into practice. A structured data abstraction form was developed to aid data collection and identify key aspects of clinical practice covered by the HIP guidance (see Additional file 1 for questions) [15]. Particular emphasis was given to assessing whether the ‘key priorities’ of the guidance were implemented. These include prescription of aspirin to women at risk of pre-eclampsia and use of an automated reagent-strip reading device or a spot urinary protein:creatinine ratio for estimating proteinuria in a secondary care setting (see Table 1). Additional questions to the key priorities were also included such as ‘was early birth offered to women with a HDP’ (early birth is birth by induction of labour or by elective caesarean section). Because this research required the collection of retrospective data without maternal consent and was conducted across several maternity units; UK Confidential Advisory Group (CAG) approval was obtained (CAG ref.: 17/CAG/0006).

**Table 1:** National Institute for health and Care Excellence’s Hypertension in Pregnancy key priority recommendations [15]

Prevention	
Advise women at high risk of pre-eclampsia to take 75 mg of aspirin daily from 12 weeks until the birth of the baby. Women at high risk are those with any of the following	
• hypertensive disease during a previous pregnancy	
• chronic kidney disease	
• autoimmune disease such as systemic lupus erythematosis or antiphospholipid syndrome	
• type 1 or type 2 diabetes	
• chronic hypertension.	
Surveillance	
Use an automated reagent-strip reading device or a spot urinary protein:creatinine ratio for estimating proteinuria in a secondary care setting (women with hypertension)	
Treatment	
Tell women who take angiotensin-converting enzyme (ACE) inhibitors or angiotensin II receptor blockers (ARBs):	
• that there is an increased risk of congenital abnormalities if these drugs are taken during pregnancy	
• to discuss other antihypertensive treatment with the healthcare professional responsible for managing their hypertension, if they are planning pregnancy.	
In pregnant women with uncomplicated chronic hypertension aim to keep blood pressure lower than 150/100 mmHg.	
Offer women with gestational hypertension and pre-eclampsia an integrated package of care covering admission to hospital, treatment, measurement of blood pressure, testing for proteinuria and blood tests as indicated	
Documentation	
Consultant obstetric staff should document in the woman’s notes the maternal (biochemical, haematological and clinical) and fetal thresholds for elective birth before 34 weeks in women with pre-eclampsia.	
Review	
Offer all women who have had pre-eclampsia a medical review at the postnatal review (6–8 weeks after the birth)	
Tell women who had pre-eclampsia their risk of developing a hypertensive disorder of pregnancy in the future	

Clinical Research Network (CRN) teams in England were informed of the research study and invited to take part via the CRN. Five maternity (four teaching and one district general) units in the Midlands and the North of England agreed to take part in the study and were provided with a random set of dates for each study year. Each team identified two to five births (depending on the number of abstractions they had agreed to make) on the dates provided and obtained the maternal records in order to abstract the information required. The CRN teams include midwives who are experienced in the abstraction of data from maternal records, training on data questions and discussion of potential issues took place prior to commencement of data abstraction. Data were inputted directly onto a secure web-based interface. To help prevent data entry errors, questions followed a logical sequence and ‘sense checks’ were applied to the web interface tool. For example, only conceivable BP levels could be inputted and only women previously identified as having hypertension could have an entry of antihypertensive medication prescribed.

### Interviews

Interviews were conducted to explore how obstetricians and midwives viewed and used the NICE HIP guidance and what they perceived were the barriers and facilitators to its use. A purposive sample of obstetricians and midwives working at the Bradford maternity unit with a range of years practicing were included. Any clinician involved in the generation of national guidance for NICE or the Royal College of midwives or Royal College of Obstetricians and Gynaecologists was excluded. Midwives were additionally selected based on their usual place of work (e.g. antenatal clinic or labour ward), because working in different settings may have influenced guidance use. Hospital management approved staff involvement in the study and all clinicians gave written consent and agreed that interviews could be recorded for the research purposes specified in the information/consent forms (Health Research Authority ethics approval is not required for research that includes NHS staff in the UK).

A topic guide was used to focus the interviews; however clinicians were able to speak freely about their experiences and raise topics. The development of the topic guide was informed by a review of relevant literature, suggestions from our multi-disciplinary study team and the findings from our evaluation of clinician adherence (see below). The topic guide was modified as data collection progressed. Clinicians were told the aim of the interview. Topics included general perceptions of the guidance, how well the guidance recommendations were implemented, personal use, understanding of the aims and perceived strengths and weaknesses and what might prevent use.

The interview was conducted in a private room within the maternity unit and started with the researcher asking for background information about the clinician, including training and work experience. Broad questions were then put to the clinician for example ‘what are your general thoughts about the NICE guidance on HIP?’ the interview then progressed according to the clinician’s responses, but also including ‘core’ questions. Interviews were conducted in the autumn of 2018 and lasted between 20 and 30 min.

### Data analysis

Quantitative data were transferred to Stata software [21] upon completion of all abstractions. ‘Before and after guidance publication’ differences in frequencies were assessed using chi-squared test and t-test for differences in mean blood pressure. Differences in proportions were also assessed using the prtesti command in Stata [21], because any differences (or similarities) in frequencies of the two groups may not accurately reflect ‘true’ differences because denominator numbers differed between the two groups.

Inductive thematic analysis was used to identify themes and patterns within the qualitative interview data. Thematic analysis offers an accessible and theoretically flexible, as well as systematic approach to analysing qualitative data [22, 23]. Interviews were transcribed in full and transcripts were read several times so that data became familiar, codes were applied and these were organised into potential themes. Themes were reviewed in relationship to the data, and then these themes were named and defined. Direct quotes were coded: obstetrician/midwife, number = participant number, this was done to protect anonymity. NVivo Version 11 qualitative analysis software (QSR International Pty Ltd) was used to categorise codes and themes.

## Results

### Data on clinical practice

Generally response percentages (when data were sufficient to provide meaningful comparison) were similar across hospitals, for the majority of questions (see Additional file 2 for individual hospital response frequencies), therefore data were combined; 2490 pregnancies were included, 1271 before NICE HIP guidance publication (2008 to September 2010) and 1219 after guidance publication (2013 to 2015) (Table 2). The proportion of women identified as being at high risk of pre-eclampsia was greater after guidance publication compared to before, though this was of borderline statistical significance; similar proportions were identified as at moderate risk. Aspirin was prescribed more frequently to both high and moderate risk women after the guidance publication compared to before. Very small numbers of women were identified as having pre-existing hypertension irrespective of time period and there was no difference in the proportion of women using ACE or ARBs in early pregnancy. Alternative medications to ACE or ARBs were prescribed more often before guidance publication, however actual numbers were small (Table 2).

**Table 2:** Prevention of pre-eclampsia and management of pre-existing hypertension. Values are n (%)

	NICE hypertension in pregnancy recommendations		*P*-value^a^	*P*-value^b^ (CI)
	Before guidance *N* = 1271	After guidance *N* = 1219		
High risk of pre-eclampsia	73 (6)	93 (8)	0.06	0.05 (−0.01, 0.04)
Moderate risk of pre-eclampsia	105 (9)	116 (10)	0.20	0.40 (−0.03, 0.01)
	*N* = 73	*N* = 93		
Aspirin prescription for high risk women	10 (14)	50 (54)	< 0.01	< 0.01 (0.37, 0.43)
	*N* = 105	*N* = 116		
Aspirin prescription for moderate risk women	3 (3)	63 (54)	< 0.01	< 0.01 (0.48, 0.54)
Management of pre-existing hypertension	*N* = 1271	*N* = 1219		
Diagnosis of pre-existing hypertension	17 (1)	41 (3)	< 0.01	< 0.01 (0.01, 0.03)
ACE or ARBs used prior to or in early pregnancy	2 (12)	10 (25)	0.30	0.27 (−0.07, 0.34)
	*N* = 2	*N* = 10		
Alternatives to ACE or ARBs prescribed	2 (100)	8 (80)	0.50	0.05 (−0.45, − 0.08)

*ACE* Angiotensin-converting enzyme, *ARB* Angiotensin receptor blockers

^a^difference in frequencies ‘before and after’ guidance publication

^b^difference in proportions ‘before and after’ guidance publication

Table 3 shows that three quarters of women had their BP recorded and a third had proteinuria estimated at every antenatal visit, both before and after guidance publication.

**Table 3:** Antenatal surveillance. Values are n (%)

	NICE hypertension in pregnancy recommendations		*P*-value^a^	*P*-value^b^ (CI)
	Before guidance *N* = 1271	After guidance *N* = 1219		
Blood pressure recorded at every antenatal visit	962 (76)	913 (75)	0.60	0.56 (−0.04, 0.02)
If not recorded at every visit how often omitted:	*N* = 309	*N* = 306	0.30	
once	217 (71)	199 (65)		0.11 (−0.14, 0.01)
twice	61 (20)	64 (21)		0.77 (−0.05, 0.07)
three	16 (5)	30 (10)		0.02 (0.01, 0.09)
four	9 (3)	7 (2)		0.43 (−0.03, 0.01)
≥ Five times	5 (2)	5 (2)		1.00 (−0.02, 0.02)
	*N* = 1271	*N* = 1219		
Proteinuria recorded at every antenatal visit	441 (35)	417 (34)	0.80	0.56 (−0.05, 0.03)
If not recorded at every antenatal visit how often omitted:	*N* = 830	*N* = 802	0.03	
once	341 (41)	315 (39)		0.41 (−0.07, 0.03)
twice	246 (30)	195 (24)		< 0.01 (−0.10, − 0.02)
three	126 (15)	147 (18)		0.10 (−0.01, 0.07)
four	64 (6)	73 (9)		0.02 (0.00, 0.06)
≥ Five times	53 (6)	70 (9)		0.02 (0.00, 0.06)

^a^difference in frequencies ‘before and after ‘guidance publication

^b^difference in proportions ‘before and after ‘guidance publication

Similar proportions of women were diagnosed with a HDP and had BP that equalled or exceeded recommended treatment thresholds during each time period (Table 4).

**Table 4:** Diagnosis and treatment. Values are n (%) unless otherwise indicated

	NICE hypertension in pregnancy recommendations		P-value^1^	P-value^2^ (CI)
	Before guidance *N* = 1271	After guidance *N* = 1219		
Gestational hypertension or pre-eclampsia^a^	227 (18)	195 (16)	0.20	0.18 (− 0.05, 0.01)
Blood pressure equal or exceeding recommended treatment threshold^b^	54 (24)	62 (32)	0.60	< 0.07 (− 0.17, 0.01)
Antihypertensive prescription (for gestational hypertension and Pre-eclampsia):	*N* = 227	*N* = 195	< 0.01	
No antihypertensive	139 (62)	53 (27)		< 0.01 (−0.44, − 0.26)
Labetalol	58 (26)	120 (62)		< 0.01 (0.27, 0.45)
Other antihypertensive prescribed^c^	28 (12)	22 (11)		0.75 (−0.07, 0.05)
Missing	2 (< 1)	–		–
Protein estimated when hypertension identified	156 (68)	148 (76)	0.80	0.07 (−0.01, 0.17)
Method used to estimate protein:	*N* = 156	*N* = 148	< 0.01	
24 h urine	13 (8)	6 (4)		0.14 (−0.09, 0.01)
Automated reagent strip reading	6 (4)	17 (11)		0.02 (0.01, 0.13)
Spot PCR	47 (30)	98 (66)		< 0.01 (0.26, 0.46)
Reagent strip visual inspection	67 (43)	26 (18)		
Other estimation	19 (12)	1 (< 1)		–
Unclear	3 (2)	–		–
Missing	1 (< 1)	–		–
Admitted due to HDP	92 (56)	127 (65)	< 0.01	< 0.001 (0.14, 0.33)
Care on hospital admission:	*N* = 92	*N* = 127		
Highest mean systolic blood pressure prior to admission (for HDP)	150	155	< 0.01 (−9.3, −1.4)	–
Highest mean diastolic blood pressure prior to admission (for HDP)	98	114	0.19 (−40.7, 8.1)	–
Protein estimated prior to admission	67 (73)	92 (72)	0.70	0.87 (− 0.13, 0.11)
Anti-hypertensive prescribed^c^	46 (50)	79 (62)	0.02	0.08 (−0.01, 0.25)
Existing hypertensive medication increased	8 (9)	19 (15)		0.18 (−0.03, 0.15)
No medication prescribed or increased	36 (39)	29 (22)		0.01 (−0.29, 0.05)
Labetalol prescribed	35 (38)	64 (50)		0.07 (−0.01, 0.25)
Labetalol increased	5 (5)	21 (17)		0.01 (0.04, 0.20)
Other antihypertensive prescribed^d^	14 (15)	13 (10)		0.26 (−0.13, 0.04)
For all women with a HDP diagnosis:	*N* = 227	*N* = 195		
Was early Birth < 37 weeks gestation offered due to HDP~	20 (9)	35 (18)	0.01	0.01 (0.02, 0.16)
If early Birth < 37 weeks gestation offered due to HDP:	*N* = 20	*N* = 35	0.20	
Was blood pressure ≥ 160/110	1 (5)	5 (14)		0.30 (−0.06, 0.24)
No evidence of BP ≥ 160/110	9 (45)	8 (23)		0.09 (−0.48, 0.04)
‘Other’ concerns	10 (50)	22 (63)		0.35 (−0.14, 0.40)
For all women with a HDP diagnosis:	*N* = 227	*N* = 195		
Was early Birth ≥ 37 weeks gestation offered due to HDP	68 (30)	103 (52)	< 0.01	< 0.01 (0.09, 0.35)
If early Birth ≥ 37 weeks gestation offered due to HDP:	*N* = 68	*N* = 103	< 0.01	
Blood pressure ≥ 160/110	8 (12)	5 (5)		0.10 (−0.16, 0.02)
No evidence of BP ≥ 160/110	54 (79)	60 (58)		< 0.01 (−0.35, − 0.07)
‘Other’ concerns	6 (9)	38 (37)		< 0.01 (0.16, 0.40)

^1^difference in frequencies ‘before and after’ guidance publication

^2^difference in proportions ‘before and after’ guidance publication

^a^diagnosis of gestational hypertension or pre-eclampsia or blood pressure equal to or greater than 140/90 on two occasions at least 4 h apart, this includes two readings at least 4 h apart with a systolic blood pressure equal to or greater than 140 with a normal diastolic or a diastolic equal to or greater than 90 with a normal systolic

^b^Blood pressure greater than or equal to recommended treatment threshold 150/100 on two occasions at least 4 h apart

^c^one or more drugs prescribed or increased

^d^methyldopa, nifedipine

Following guidance publication when hypertension was identified, more women were prescribed an anti-hypertensive and more often that antihypertensive was labetalol. The estimation of protein did not increase after guidance publication, however when it was estimated a spot protein:creatinine ratio (PCR) was more frequently the method of choice (Table 4).

Table 4 also shows that women were more frequently admitted to hospital because of their HDP following guidance publication. Prior to their admission due to a HDP, mean DBP was similar across time periods, however following guidance publication mean SBP was significantly higher. There was no difference in the frequency of protein estimation prior to admission due to a HDP across time periods. When admitted, antihypertensives were similarly prescribed or dose increased across time periods, however before guidance publication more women received no medication or medication increase. After guidance when admitted due to a HDP the drug of choice was more frequently labetalol.

Table 4 shows that when women were offered early birth because of their HDP (early birth by induction of labour or by elective caesarean section if indicated) at gestations < 37 and ≥ 37 weeks, the proportions of women with BP ≥ 160/110 were similar across time periods. After guidance publication the proportion of women offered early birth at ≥ 37 gestation with BP less than 160/110 were fewer and more had other concerns documented compared to women before guidance publication.

The proportion of women who experienced a HDP that were informed of their increased risk of developing a HDP in any future pregnancy was greater following guidance publication. Most women who had developed a HDP were advised to see their GP for their postnatal review both before and after guidance publication, though the proportion increased significantly after guidance publication (Table 5).

**Table 5:** Postnatal management for women diagnosed with a HDP. Values are n (%)

	NICE hypertension in pregnancy guidance		*P*-value^a^	*P*-value^b^ (CI)
	Before guidance *N* = 227	After guidance *N* = 195		
Informed of increased risk of HDP in future pregnancy	4 (2)	20 (10)	< 0.01	< 0.01 (0.03, 0.13)
Medical review conducted at the postnatal review	13 (6)	9 (5)	< 0.01	0.65 (− 0.05, 0.03)
Unknown-advised to see GP for postnatal review	132 (58)	176 (90)		< 0.01 (0.24, 0.39)
No evidence	81 (36)	10 (5)		< 0.01 (− 0.38, − 0.24)

^a^difference in frequencies ‘before and after’ guidance publication

^b^difference in proportions ‘before and after’ guidance publication

### Interviews

Eleven clinicians; five obstetricians and six midwives with a range of practicing years (obstetricians, 10–27 years, midwives, 5–20 years) were interviewed. Obstetricians worked across all hospital settings as did midwives, however all the obstetricians spent several hours during each day in one or more setting, whereas midwives spent several months/years working in one setting, for example labour ward or antenatal clinic. Examples of responses are provided below. Overall clinicians viewed the NICE HIP guidance favourably; they said recommendations were supported by robust evidence and that care had improved and become more standardised following guidance publication. However, most clinicians acknowledged that the guidance was lengthy, sometimes non-specific and not always adhered to. Four general themes were identified.

#### Usefulness of NICE guidance

All the clinicians thought that NICE guidance was an important resource; all said that NICE guidance informed their local hospital guidance document: *“I think we do mould our practice according to NICE guidance, it’s good to have a national standard” (obstetrician 1, 27 years practice) and “I think it was clear, well received, lots of good clear steers on what tests to do and when to repeat” (obstetrician 3, 19 years practice) and “I think the guidance works, the women get a good standard of care, close monitoring of BP [blood pressure], tweaking of medication, we use the standard medications labetalol and nifedipine, its very rare we use alternative ones” (midwife 4, 10 years practice).*

#### Quality of underpinning evidence

All clinicians thought that the guidance was informed by robust evidence. *“They [NICE guidance development group] are a robust body of respected opinion leaders that come up with these recommendations that you know will be founded on solid research evidence, they [guidance recommendations] are always going to be well respected, well thought through” (obstetrician 1, 27 years practice) and “Basically they’ve done the work for you and they have the most up to date information to inform practice” (midwife 5, 8 years practice).*

#### NICE and local guidance

Clinicians generally accessed their local guidance for information initially, stating that their local guidance was based on NICE guidance, was simplified (compared to lengthy NICE guidance) and easy to access. However if more in-depth information was required they spent time finding the information and reading through NICE guidance: “*I tend to use the local guidance, I tend to check a lot because when I was on the birth centre I didn’t look at the high risk stuff, but now I’m on the ward I probably access [guidance] more because I’m trying to familiarise myself” (Midwife 5, 8 years practice) and “I rarely use NICE guidance I most often go to local guidance which is based on NICE” (obstetrician 3, 19 years practice).”I go to local guidance first they are easy to access [there is an icon on the computer] and short, so when you’re in a busy clinic that’s great, but if I want to know something that isn’t in our guideline I go to NICE” (Midwife 3, 20 years practice).*

#### Barriers to NICE HIP guidance use and adherence

Although all clinicians thought the NICE guidance was a useful resource, most said it was lengthy and that finding definitions and specific practice recommendations could be challenging and that sometimes evidence may be out of date. Several acknowledged that the NICE guidance is not adhered to all of the time: *“Not updated so often may mean they are out of date, therefore people do their own thing, can be lengthy and sometimes I don’t agree with their recommendations so sometimes we choose to not do some of the things or [we] do extra things” (obstetrician 2, 10 years practice) “It can take time to find specific recommendations, in a busy clinic that is a problem, the volume of info is large but the flow charts help with the treatment thresholds and recommendations” (obstetrician 4, 12 years practice*). *“Clinics can be really busy so making women understand the importance of recognising and reporting symptoms or waiting for urine specimens isn’t sometimes possible, you have to prioritise, provide some care to all women and try and not hold things up too much” (midwife 1, 5 years practice). I think where we don’t adhere is the gestational hypertension … ………… … … I think people get too many blood tests … .. over brought back, over treated (obstetrician 2, 10 years practice,)* the same obstetrician said *“a draft of our local guidance has been sent around and it suggests we manage pre-eclampsia as an outpatient, which is not what NICE recommend”.*

## Discussion

Our findings suggest that the publication of the NICE HIP guidance has influenced some important aspects of care including the provision of prophylactic aspirin, which has been shown to reduce the risk of pre-eclampsia [24] and the use of labetalol to control high blood pressure [25]. The NICE guidance recommendations include BP thresholds for the diagnosis of HDP; although the detection of HDP did not change across the study period it is possible that guidance threshold recommendations influenced HDP classification because mean systolic blood pressure prior to admission for a HDP was significantly higher following publication, which may reflect the NICE guidance recommendation to only admit women to hospital with severe gestational hypertension (160/110 mmHg or higher) or pre-eclampsia (irrespective of the degree of hypertension). Although this seems a less conservative approach, more intensive surveillance can be reserved for women most at risk of an adverse outcome and most likely to benefit, which is important at a time when many maternity units are under increasing pressures.

NICE recommend early birth (by induction of labour or by elective caesarean section) for women with pre-eclampsia, before 37 weeks depending on maternal and fetal condition and response to treatment and at or after 37 weeks when hypertension is mild to moderate [15, 17]. Early birth before 37 weeks was offered more frequently to women and there were ‘other concerns’ more frequently documented after guidance publication. Reasons for this are unclear, but may reflect improving documentation of management decisions or a greater awareness of the implications of preterm birth (and therefore an improved understanding that offering early birth may increase some harms whilst providing some benefits). There may also have been improved BP monitoring of women with a HDP after guidance publication (and therefore increased likelihood of identifying a problem). Although the highest mean Systolic BP prior to admission was statistically significantly higher, mean Diastolic BP was similar following guidance publication, the difference was still relatively small and therefore would not likely influence clinical decision making in a meaningful way.

Some aspects of care seem unaffected by NICE guidance recommendations, particularly estimation of proteinuria at routine antenatal assessments and also at the time hypertension was identified, though the use of more objective tests; the spot protein:creatinine ratio and automated reagent strip reading device did increase following guidance publication. These objective measurements are important for the correct identification of pre-eclampsia. Even though only one in every million women in the UK dies from a HDP [26], globally an estimated 29,000 women die each year from a HDP, if health care was available to these women and HDPs identified and treated appropriately, the majority of these deaths could be prevented [27–29]. Indeed in the UK and Ireland five of the 14 women who died from a HDP between 2009 and 2014 had not had their blood pressure measured at their initial antenatal care visit, and several did not have their urine checked at each subsequent visit [26]. We found that blood pressure was usually measured, possibly because it is less troublesome to obtain compared to a urine sample. When clinicians were interviewed and asked about barriers to guidance adherence and why protein estimation may be inconsistently estimated (compared to blood pressure) they suggested it may be due to inadequate toilet facilities, overly busy clinics (not allowing for the time needed to produce a urine specimen) and lack of compliance from the women. There could also be a lack of understanding (by some clinicians) regarding the importance of proteinuria estimation, and therefore an acceptance by clinicians of the ‘no specimen available’ scenario that seems often recorded in maternal records, however the clinicians we interviewed were aware of the importance of proteinuria estimation.

We found that although more women were informed of their increased risk of developing a hypertensive disorder in a future pregnancy, the number was small even after guidance publication, this may improve following the recent guidance update because the recommendation has been simplified from indicating ranges of future risk by each HDP to “advise women with hypertensive disorders of pregnancy that the overall risk of recurrence in future pregnancies is approximately 1 in 5” [17]. The majority of women were advised to see their general practitioner (GP) for their 6 week postnatal review, so predictably we found few women who had developed a HDP with documented evidence of a medical review in their maternity records and this did not change following guidance publication; however that is not to say that they did not receive a medical review from their GP. The updated guidance recommends that women who have developed a HDP should be offered a medication review at 2 weeks (if on antihypertensives) and medical review at six to 8 weeks, either with their GP or specialist. Women should be informed of the importance of attending a review and encouraged to attend their GP or specialist because a significant proportion will remain hypertensive following birth [30] and having developed a hypertensive disorder in pregnancy (irrespective of BP level following birth) is associated with an increased risk in all-cause mortality [7], Alzheimer’s disease, diabetes, ischaemic heart disease and stroke [5–7]. The postnatal review is therefore an important opportunity to discuss future health; offer continuing surveillance and provide advice on behaviour changes that may help reduce longer-term risk of disease development.

### Strengths and limitations

We were able to include ~2500 pregnancies using a random selection of birth dates from five English maternity units; we included 6 years of practice behaviour to establish the extent to which the NICE HIP guidance influenced clinical care, making our study’s findings generalisable to other English maternity units. The data were abstracted by trained and experienced CRN teams and inputted directly onto a web interface that was designed specifically for this activity. Due of data protection legislation and because it was not possible to gain consent from women whose records were accessed (because they were no longer under the care of maternity services), data were not independently scrutinised for accuracy. A retrospective maternity records review was required, rather than using routinely collected hospital data because generally, the information about adherence to recommendations was not routinely collected. Maternity units should therefore consider collecting such data so that evaluating care practices can be more easily achieved.

NICE updated the HIP guidance in June 2019 [17], however most recommendations we assessed in our study have not changed substantially and therefore our findings still provide an appropriate estimate of clinical adherence. There are some new HIP recommendations and modifications to others, for example the estimation of placental growth factor (PIGF) if there is suspicion of pre-eclampsia and target BP which we could not assess [17].

Limitations of our study include the retrospective design; for example it was not possible to ask clinicians about their decisions or find missing data that may be possible in a prospective study. A before and after design does not provide robust evidence of causality thus it is impossible to say whether the changes we found were wholly attributable to NICE guidance publication as it is possible that changes in care would have happened without the NICE guidance. However the response to our interview questions help to make us more confident that change in practice reflected the influence of the guidance. The maternity unit CRN teams taking part were self-selected; care provided in their maternity units may therefore differ from the maternity units whose CRN teams decided not to take part, however we conducted a before and after assessment of clinical care within these maternity units, therefore our findings are valid. Rates of hypertension and pre-eclampsia in our study are higher than would be expected in most UK populations. We particularly wanted to evaluate the care of women with a HDP (as well as those without), therefore there may have been a bias towards the inclusion of women with a HDP if more than the required number of births occurred on any of the random dates, this would have happened across all abstraction years (and we report similar HDP rates across the two periods supporting this suggestion) so this would not influence the differences (or similarities) following guidance publication we report. We also asked teams to include a woman as having gestational hypertension or pre-eclampsia, irrespective of the blood pressure reading if there was documented evidence of a HDP diagnosis, therefore the higher rate may also reflect our relatively more inclusive criteria for a HDP compared with some studies that may have used tighter criteria.

It has been previously suggested that guidance recommendations may be more likely to be adopted when there is strong professional support, a stable and convincing evidence base, and no increased or unfunded costs [31]. Costs were not considered in this research however our interviews suggest strong professional support for the NICE HIP guidance and an acknowledgement of the robust evidence that underpins the recommendations.

We interviewed clinicians from one maternity unit regarding their use and perceptions of the NICE HIP guidance which may limit generalisability to other clinicians; however all clinicians had trained and worked at other hospitals or were still in training, so moved from hospital to hospital. They also discussed general guidance use across the whole of their practice years rather than only in their current position.

## Conclusions

The publication of the NICE HIP guidance has influenced important aspects of care that may help improve outcomes for women who have pre-existing hypertension or who develop a hypertensive disorder of pregnancy. We found that clinicians view NICE guidance favourably and believe the recommendations are underpinned by robust evidence, which may increase the likelihood of recommendation adherence. Even so some aspects of care such as proteinuria estimation seem unaffected by guidance recommendations, suggesting work is needed to increase understanding amongst clinicians regarding those issues.

## Supplementary information

**Additional file 1.** Survey Questions.

**Additional file 2 : Table S1**. Prevention of pre-eclampsia and management of pre-existing hypertension by hospital (A-E). Values are n (%) of the included/eligible hospital population. **Table S2**. Antenatal surveillance by hospital (A-E). Values are n (%) of the included/eligible hospital population. **Table S3**. Diagnosis and treatment. Values are n (%) of the included/eligible hospital population. **Table S4**. Postnatal management of women diagnosed with a HDP. Values are n (%) of the included/eligible hospital population.

## Data Availability

Data are available from the corresponding author upon request.

## Abbreviations

ACE: Angiotensin-converting enzyme
ARB: Angiotensin receptor blockers
BP: Blood pressure
CAG: Confidential advisory group
CI: Confidence interval
CRN: Clinical research network
GH: Gestational hypertension
GP: General practitioner
HDP: Hypertensive disorders of pregnancy
HIP: Hypertension in pregnancy
NICE: National Institute for Health and Care Excellence
PE: Pre-eclampsia
PIGF: Placental growth factor
UK: United Kingdom
