# Supplementary material for: An evaluation of the influence of the publication of the UK National Institute for health and Care Excellence’s guidance on hypertension in pregnancy: a retrospective analysis of clinical practice
Source: BMC Pregnancy Childbirth. 2020 Feb 12;20:101. doi: 10.1186/s12884-020-2780-y (PMC7017474; doi:10.1186/s12884-020-2780-y)
Supplement: Supplementary file 1 — Additional file 1. Survey Questions. [file 12884_2020_2780_MOESM1_ESM.pdf]

**Survey Questions:**		
A00a & A00b		**Infant's month and year of birth**
A001-A004	Prevention	**If any one (or more) risk factor below is present this signifies high risk and need for aspirin**<br>• hypertensive disease (gestational hypertension/pre-eclampsia) during a previous pregnancy<br>• chronic kidney disease<br>• autoimmune disease such as systemic lupus erythematosis or antiphospholipid syndrome<br>• type 1 or type 2 diabetes<br>• chronic hypertension.<br><br>**If any two moderate risk factors are present this signifies moderate risk and need for aspirin**<br>• first pregnancy<br>• age 40 years or older<br>• pregnancy interval of more than 10 years<br>• body mass index (BMI) of 35 kg/m² or more at first visit<br>• family history of pre-eclampsia<br>• multiple pregnancy.
B001-B003	Surveillance	Hypertension that is present at the booking visit or before 20 weeks (140/90) or if the woman is taking antihypertensive medication when pregnancy is diagnosed. The raised BP can be primary or secondary to another condition.
B001-B003	Surveillance	ACE or ARBs are not recommended for use in pregnancy therefore alternatives should be prescribed as soon as pregnancy is confirmed<br><br>**Angio converting enzyme (ACE) inhibitors**<br>Benazepril - Lotensin<br>Captopril - Capoten<br>Enalapril - Vasotec, Epaned<br>Fosinopril - Monopril<br>Lisinopril - Prinivil, Zestril<br>Moexipril - Univasc<br>Perindopril - Aceon<br>Quinapril - Accupril<br>Ramipril - Altace<br>trandolapril - Mavik<br>**Angiotensin receptor blockers (ARBs)**<br>Candesartan - Amias<br>Eprosartan - Teveten<br>Irbesartan - Aprovel, CoAprovel<br>Losartan - Cozaar, Cozaar Comp<br>Olmesartan - Olmetec, Olmetec Plus<br>Telmisartan - Micardis, Micardis Plus<br>Valsartan - Diovan, Co-Diovan, Exforge
B004-B007	Surveillance	Refers to each separate antenatal visit that necessitates an 'antenatal assessment', it could be that a woman was seen on the same day more than once, so each admission to the maternity assessment unit, each antenatal day unit visit or each community

		clinic or a home visit, irrespective of the reason for the visit
B008-B009	Surveillance	If a woman with hypertension was seen within the maternity unit (secondary care facility) was there evidence that an **initial** automated reagent-strip reading device was used or evidence that a urine specimen was sent to the lab for protein: creatinine ratio (PCR) estimation for each antenatal visit/assessment<br><br>(automated reagent –strip reading device use is not recommended for women seen in primary care facilities (GP surgeries for example) please do not include assessments conducted in primary care in this section)<br><br>If automated reagent-strip reading device showed equal to or more than +1 protein, was a urine sample sent to the biochemistry laboratory for protein: creatinine ratio (PCR) estimation
C001	Diagnosis and treatment	Is there evidence this woman had gestational hypertension (BP equal to or greater than 140/90 on two occasions at least 4 hours apart, this includes two readings at least 4 hours apart with a systolic BP equal to or greater than 140 with a normal diastolic or a diastolic equal to or greater than 90 with a normal systolic) with or without proteinuria
C002	Diagnosis and treatment	Did BP ever reach or exceed the recommended treatment threshold 150/100 two occasions at least 4 hours apart
C003	Diagnosis and treatment	If this woman was diagnosed with gestational hypertension (irrespective of BP level), what treatment was started
C004	Diagnosis and treatment	If the women was diagnosed with gestational hypertension (irrespective of BP level), was proteinuria estimated at that time
C005	Diagnosis and treatment	If the woman had proteinuria (at the time her BP exceeded the threshold for treatment) what method was used to estimate the proteinuria (tick all that apply)
C006	Diagnosis and treatment	Was the woman ever admitted to hospital because of high blood pressure (antenatal admission only)
C007-C008	Diagnosis and treatment	If this woman was admitted to hospital because of high blood pressure antenatally, what was her highest blood pressure prior to admission (the highest diastolic and systolic readings may not be recorded together, i.e. may have occurred at different times)
C008	Diagnosis and treatment	If admitted to hospital antenatally, what was the protein estimation prior to admission
C009	Diagnosis and treatment	If admitted because of high blood pressure were antihypertensive medications prescribed
C009	Diagnosis and treatment	If antihypertensive medications were prescribed when admitted because of high blood pressure, which medication (s) was/were prescribed
D001	Timing of birth	Was early delivery (**before 37 weeks**) offered because of hypertension (induction or caesarean section)
D002	Timing of birth	If early birth offered (**before 37 weeks**) was BP generally above 160/110

D003	Timing of birth	Was early delivery (**after 37 weeks**) offered because of hypertension (induction or caesarean section)
D004	Timing of birth	If early birth offered (**after 37 weeks**) was BP generally above 160/110
E001	Postnatal follow-up	If diagnosed with hypertension or pre-eclampsia, was there any evidence that future risk of gestational hypertension and pre-eclampsia was discussed
E002	Postnatal follow-up	Was there any evidence that a postnatal review appointment was given
E003	Postnatal follow-up	If there was evidence that the woman attended a postnatal review (6–8 weeks after the birth) was a medical review within that appointment recorded
