# Supplementary material for: An evaluation of the influence of the publication of the UK National Institute for health and Care Excellence’s guidance on hypertension in pregnancy: a retrospective analysis of clinical practice
Source: BMC Pregnancy Childbirth. 2020 Feb 12;20:101. doi: 10.1186/s12884-020-2780-y (PMC7017474; doi:10.1186/s12884-020-2780-y)
Supplement: Supplementary file 2 — Additional file 2 : Table S1. Prevention of pre-eclampsia and management of pre-existing hypertension by hospital (A-E). Values are n (%) of the included/eligible hospital population. Table S2. Antenatal surveillance by hospital (A-E). Values are n (%) of the included/eligible hospital population. Table S3. Diagnosis and treatment. Values are n (%) of the included/eligible hospital population. Table S4. Postnatal management of women diagnosed with a HDP. Values are n (%) of the included/eligible hospital population. [file 12884_2020_2780_MOESM2_ESM.pdf]

Supplementary table 1 Prevention of pre-eclampsia and management of pre-existing hypertension by hospital (A-E). Values are n (%) of the included/eligible hospital population

	**NICE hypertension in pregnancy recommendations**	
	**Before guidance**	**After guidance**
High risk of pre-eclampsia		
A	6 (4)	5 (3)
B	33 (9)	15 (4)
C	14 (9)	37 (23)
D	11 (3)	19 (5)
E	9 (4)	17 (9)
Moderate risk of pre-eclampsia		
A	9 (6)	6 (4)
B	42 (12)	27 (8)
C	10 (7)	12 (10)
D	39 (11)	64 (18)
E	5 (3)	7 (4)
Aspirin prescription for high risk women		
A	1 (17)	3 (60)
B	2 (6)	8 (53)
C	3 (21)	16 (43)
D	3 (27)	17 (89)
E	1 (11)	6 (35)
Aspirin prescription for moderate risk women		
A	-	-
B	-	9 (33)
C	-	4 (33)
D	2 (5)	49 (77)
E	1 (20)	1 (14)
Diagnosis of pre-existing hypertension		
A	-	-
B	3 (1)	1 (<1)
C	6 (4)	10 (6)
D	8 (2)	25 (7)
E	-	5 (3)
ACE or ARBs used prior to or in early pregnancy		
B	-	-
C	-	-
D	2 (33)	4 (40)
E	-	6 (25)
B	-	-
Alternatives to ACE or ARBs prescribed		
A	-	-
B	-	-
C	2 (100)	4 (100)
D	-	4 (67)
E	-	-

ACE = Angiotensin-converting enzyme

ARB = Angiotensin receptor blockers

Supplementary table 2 Antenatal surveillance by hospital (A-E). Values are n (%) of the included/eligible hospital population

	**NICE hypertension in pregnancy recommendations**	
	**Before guidance**	**After guidance**
Blood Pressure recorded at every antenatal visit		
A	92 (61)	110 (74)
B	340 (90)	299 (87)
C	89 (55)	105 (65)
D	260 (70)	269 (72)
E	181 (87)	130 (68)
If not recorded at every visit how often omitted		
once		
A	42 (72)	26 (67)
B	29 (76)	25 (56)
C	53 (73)	37 (66)
D	72 (64)	68 (64)
E	21 (77)	43 (72)
twice		
A	13 (22)	9 (23)
B	6 (16)	10 (22)
C	11 (15)	14 (25)
D	26 (23)	19 (18)
E	5 (19)	12 (20)
three		
A	2 (3)	4 (10)
B	2 (5)	8 (18)
C	7 (10)	3 (5)
D	5 (4)	12 (11)
E	-	3 (5)
four		
A	1 (2)	0 (-)
B	1 (3)	2 (4)
C	1 (1)	1 (1)
D	6 (5)	3 (3)
E	-	1 (2)
$\geq$Five times		
A	-	-
B	-	-
C	1 (1)	-
D	3 (3)	4 (4)
E	1 (4)	-
Proteinuria recorded at every antenatal visit		
A	17 (11)	30 (20)
B	122 (32)	89 (26)
C	36 (22)	31 (19)
D	218 (59)	198 (53)
E	48 (23)	69 (36)
If not recorded at every antenatal visit how often omitted		
once		
A	38 (29)	49 (42)
B	77 (30)	37 (16)
C	68 (54)	63 (48)
D	96 (62)	116 (66)
E	62 (39)	50 (42)
twice		

	NICE hypertension in pregnancy recommendations	
	Before guidance	After guidance
A	31 (23)	31 (26)
B	96 (38)	68 (27)
C	35 (28)	35 (27)
D	38 (25)	28 (16)
E	46 (29)	33 (28)
three		
A	26 (20)	22 (19)
B	47 (18)	77 (30)
C	17 (13)	18 (14)
D	10 (6)	17 (10)
E	26 (16)	13 (11)
four		
A	17 (13)	8 (7)
B	21 (8)	39 (15)
C	6 (5)	9 (7)
D	6 (4)	5 (3)
E	14 (9)	12 (10)
≥Five times		
A	21 (16)	8 (7)
B	15 (6)	34 (13)
C	1 (<1)	5 (4)
D	4 (3)	11 (6)
E	12 (8)	12 (10)

Supplementary table 3 Diagnosis and treatment. Values are n (%) of the included/eligible hospital population

	**NICE hypertension in pregnancy recommendations**	
	**Before guidance**	**After guidance**
Gestational hypertension or pre-eclampsia[a]		
A	14 (9)	9 (6)
B	92 (24)	55 (34)
C	18 (11)	24 (6)
D	91 (24)	97 (26)
E	12 (6)	10 (5)
Blood pressure equal or exceeding recommended treatment threshold[b]		
A	3 (21)	2 (22)
B	9 (10)	13 (24)
C	7 (40)	5 (21)
D	33 (36)	37 (38)
E	2 (17)	5 (50)
**Antihypertensive prescription (for gestational hypertension and Pre-eclampsia):**		
No antihypertensive		
A	9 (64)	2 (22)
B	81 (89)	11 (20)
C	6 (33)	18 (75)
D	37 (41)	21 (22)
E	6 (50)	1 (10)
Labetalol		
A	5 (36)	7 (78)
B	9 (10)	40 (73)
C	10(50)	6 (25)
D	29 (32)	59 (60)
E	5 (42)	8 (80)
Other antihypertensive prescribed[c]		
A	-	-
B	1 (1)	4 (7)
C	2 (11)	-
D	24 (27)	17 (18)
E	1 (8)	1 (10)
Missing		
A	-	-
B	1 (1)	-
C	-	-
D	1 (1)	-
E	-	-
Protein estimated when hypertension identified		
A	13 (93)	8 (89)
B	59 (65)	52 (35)
C	12 (67)	21 (88)
D	61 (67)	58 (60)
E	10 (83)	9 (90)
**Method used to estimate protein:**		
24 hour urine		
A	-	-
B	1 (2)	-
C	8 (67)	-
D	3 (5)	6 (10)

	NICE hypertension in pregnancy recommendations	
	Before guidance	After guidance
E	1 (10)	-
Automated reagent strip reading		
A	-	4 (50)
B	-	12 (23)
C	-	-
D	6 (10)	1 (2)
E	-	-
Spot PCR		
A	2 (15)	3 (38)
B	7 (12)	34 (65)
C	-	11 (52)
D	37 (61)	45 (46)
E	1 (10)	5 (56)
Reagent strip visual inspection		
A	11 (85)	1 (13)
B	32 (54)	6 (12)
C	4 (33)	10 (48)
D	12 (20)	5 (9)
E	8 (80)	4 (44)
Other estimation		
A	-	-
B	19 (32)	-
C		-
D	-	1 (2)
E	-	-
Unclear		
A	-	-
B	-	-
C	-	-
D	3 (5)	-
E	-	-
Missing		
A	1 (8)	-
B	-	-
C	-	-
D	-	-
E	-	-
Admitted due to HDP		
A	5 (36)	6 (67)
B	14 (15)	12 (50)
C	16 (89)	42 (76)
D	50 (56)	65 (67)
E	7 (58)	2 (20)
**Care on hospital admission:**		
Highest mean systolic BP prior to admission (for HDP)		
A	157	150
B	150	153
C	150	151
D	150	159
E	146	160
Highest mean diastolic BP prior to admission (for HDP)		
A	103	98
B	98	109
C	98	133
D	97	101

	NICE hypertension in pregnancy recommendations	
	Before guidance	After guidance
E	97	112
Protein estimated prior to admission		
A	5 (100)	6 (100)
B	13 (93)	11 (92)
C	7 (44)	31 (82)
D	40 (80)	42 (65)
E	2 (73)	2 (100)
Anti-hypertensive prescribed[c]		
A	5 (100)	4 (67)
B	6 (43)	4 (33)
C	8 (50)	27 (64)
D	24 (50)	42 (65)
E	3 (43)	2 (100)
Existing hypertensive medication increased		
A	-	1 (17)
B	1 (7)	2 (17)
C	3 (19)	8 (19)
D	4 (8)	8 (12)
E	-	-
No medication prescribed or increased		
A	-	1 (17)
B	7 (50)	6 (50)
C	5 (31)	7 (17)
D	20 (42)	15 (23)
E	4 (57)	-
Labetalol prescribed		
A	5 (100)	4 (80)
B	6 (86)	2 (33)
C	6 (55)	25 (71)
D	16 (57)	32 (64)
E	2 (67)	1 (50)
Labetalol increased		
A	-	1 (20)
B	1 (14)	4 (67)
C	1 (9)	8 (23)
D	3 (11)	7 (14)
E	-	1 (50)
Other antihypertensive prescribed[d]		
A	-	-
B	-	-
C	4 (36)	2 (6)
D	9 (32)	11 (22)
E	1 (33)	-
**For all women with a HDP diagnosis:**		
Was early Birth <37 weeks gestation offered due to HDP~		
A	2 (14)	1 (13)
B	-	4 (17)
C	9 (50)	14 (25)
D	8 (9)	15 (15)
E	1 (8)	1 (10)
**If early Birth <37 weeks gestation offered due to HDP:**		
Was BP $\geq$160/110		
A	1 (50)	-
B	-	-
C	-	1 (7)

	NICE hypertension in pregnancy recommendations	
	**Before guidance**	**After guidance**
D	-	3 (20)
E	1 (5)	1 (100)
No evidence of BP≥160/110		
A	1 (50)	1 (100)
B	4 (44)	2 (50)
C	4 (50)	3 (21)
D	-	2 (13)
E	9 (45)	-
'Other' concerns		
A	-	-
B	-	2 (50)
C	5 (56)	10 (71)
D	4 (50)	10 (67)
E	1 (100)	-
Was early Birth ≥37 weeks gestation offered due to HDP		
A	4 (33)	6 (86)
B	19 (21)	9 (45)
C	4 (44)	33 (80)
D	34 (41)	51 (62)
E	7 (64)	4 (44)
**If early Birth ≥37 weeks gestation offered due to HDP:**		
Was BP≥160/110		
A	-	-
B	1 (5)	-
C	1 (25)	-
D	6 (18)	5 (10)
E	-	-
No evidence of BP≥160/110		
A	3 (75)	3 (50)
B	18 95)	9 (100)
C	3 (75)	17 (52)
D	23 68()	30 (59)
E	7 (100)	1 (25)
'Other' concerns		
A	1 (5)	3 (50)
B	-	-
C	-	16 (48)
D	5 (15)	16 (31)
E	-	3 (75)

[a]diagnosis of gestational hypertension or pre-eclampsia or BP equal to or greater than 140/90 on two occasions at least 4 hours apart, this includes two readings at least 4 hours apart with a systolic BP equal to or greater than 140 with a normal diastolic or a diastolic equal to or greater than 90 with a normal systolic

[b]BP greater than or equal to recommended treatment threshold 150/100 on two occasions at least 4 hours apart

[c]one or more drugs prescribed or increased

[d]methyldopa, nifedipine

Supplementary table 4 Postnatal management of women diagnosed with a HDP. Values are n (%) of the included/eligible hospital population

	NICE hypertension in pregnancy guidance	
	**Before guidance**	**After guidance**
Informed of increased risk of HDP in future pregnancy		
A	-	-
B	1 (1)	-
C	2 (11)	9 (16)
D	1 (1)	9 (9)
E	-	2 (20)
Medical review conducted at the postnatal review		
A	-	-
B	-	-
C	8 (44)	4 (7)
D	3 (3)	4 (4)
E	2 (16)	1 (10)
Unknown- advised to see GP for postnatal review		
A	9 (64)	9 (100)
B	24 (26)	19 (79)
C	9 (50)	50 (91)
D	82 (91)	89 (92)
E	8 (67)	9 (90)
No evidence		
A	5 (36)	-
B	68 (74)	5 (21)
C	1 (6)	1 (2)
D	5 (6)	4 (4)
E	2 (17)	-

HDP=hypertensive disorder of pregnancy
